# Highly sensitive and specific novel biomarkers for the diagnosis of transitional bladder carcinoma

**DOI:** 10.18632/oncotarget.3841

**Published:** 2015-04-15

**Authors:** Prashant Kumar, Sayantani Nandi, Tuan Zea Tan, Siok Ghee Ler, Kee Seng Chia, Wei-Yen Lim, Zentia Bütow, Dimitrios Vordos, Alexandre De laTaille, Muthafar Al-Haddawi, Manfred Raida, Burkhard Beyer, Estelle Ricci, Marc Colombel, Tsung Wen Chong, Edmund Chiong, Ross Soo, Mi Kyoung Park, Hong Koo Ha, Jayantha Gunaratne, Jean Paul Thiery

**Affiliations:** ^1^ Institute of Molecular and Cell Biology, Agency for Science, Technology and Research (A*STAR), Singapore; ^2^ Cancer Science Institute, National University of Singapore, Singapore; ^3^ Quantitative Proteomics Group, Institute of Molecular and Cell Biology, Agency for Science, Technology and Research, Singapore; ^4^ Department of Anatomy, Yong Loo Lin School of Medicine, National University of Singapore, Singapore; ^5^ Saw Swee Hock School of Public Health, National University of Singapore, Singapore; ^6^ CHU Hopital Henri Mondor, Department of Urology, Créteil, France; ^7^ Department of Biochemistry, Yong Loo Lin School of Medicine, National University of Singapore, Singapore; ^8^ Department of Urology, Section for Translational Prostate Cancer Research, University Medical Center Hamburg-Eppendorf, Hamburg, Germany; ^9^ Service d'Urologie et Chirurgie de la Transplantation, Hôpital Edouard Herriot, Lyon, France; ^10^ Department of Urology, Singapore General Hospital, Singapore; ^11^ Department of Urology, University Surgical Cluster, National University Health System (NUHS), Singapore; ^12^ Haematology-Oncology Research Group, Department of Haematology-Oncology, National University Cancer Institute (NCIS), National University Hospital, Singapore; ^13^ Institute of Microelectronics (IME), A*STAR, Singapore; ^14^ Department of Urology, Pusan National University Hospital, Pusan National University School of Medicine, Busan, Korea

**Keywords:** transitional bladder carcinoma, urine biomarkers, receiving operating characteristics, combination model

## Abstract

Transitional bladder carcinoma (BCa) is prevalent in developed countries, particularly among men. Given that these tumors frequently recur or progress, the early detection and subsequent monitoring of BCa at different stages is critical. Current BCa diagnostic biomarkers are not sufficiently sensitive for substituting or complementing invasive cystoscopy. Here, we sought to identify a robust set of urine biomarkers for BCa detection. Using a high-resolution, mass spectrometry-based, quantitative proteomics approach, we measured, compared and validated protein variations in 451 voided urine samples from healthy subjects, non-bladder cancer patients and patients with non-invasive and invasive BCa. We identified five robust biomarkers: Coronin-1A, Apolipoprotein A4, Semenogelin-2, Gamma synuclein and DJ-1/PARK7. In diagnosing Ta/T1 BCa, these biomarkers achieved an AUC of 0.92 and 0.98, respectively, using ELISA and western blot data (sensitivity, 79.2% and 93.9%; specificity, 100% and 96.7%, respectively). In diagnosing T2/T3 BCa, an AUC of 0.94 and 1.0 was attained (sensitivity, 86.4% and 100%; specificity, 100%) using the same methods. Thus, our multiplex biomarker panel offers unprecedented accuracy for the diagnosis of BCa patients and provides the prospect for a non-invasive way to detect bladder cancer.

## INTRODUCTION

Transitional bladder carcinoma (BCa) is the fifth-most common cancer and results in significant morbidity and mortality. Cystoscopy and voided urine cytology are still the gold standards for the detection and follow-up of BCa [1] but they cannot detect certain lesions, such as small carcinoma *in situ*, and are often employed whenever patients present with other clinical signs. BCa exhibits significant tumor heterogeneity, reflected by multiple genetic alterations and complex somatic mutational profiles [2]. New biomarkers, based on DNA methylation profiling [3], point mutations [4] and microRNAs [5], have been established in addition to many proposed protein biomarkers.

Commercial tests that detect Nuclear Matrix Protein 22 (NMP22) [6] or Bladder Tumor Antigen (BTA) [7] are FDA-approved tests for BCa diagnosis, but these single-marker assays lack the specificity of voided urine cytology [8]. In addition, two protein signatures comprising ten [9] or eight [10] biomarkers were developed more recently for the detection of recurrent BCa. However, none of these diagnostic marker assays offer sufficient sensitivity and specificity to be routinely used in the clinic. It is thus desirable to identify an optimal number of highly specific biomarkers that, in combination, can improve the robustness of the currently available diagnostic methodologies.

Advanced mass spectrometry (MS)-based quantitative proteomics has emerged as a powerful technology that delivers accurate and unbiased information about the quantitative behavior of a wide variety of proteins in complex biological samples. Thus, the aim of our study was to establish a multi-analyte assay for non-muscle invasive (Ta/T1) and muscle invasive (T2/T3) BCa detection in urine using MS technology in combination with multiplex peptide stable isotope labeling (reductive dimethyl labeling strategy)-a novel approach for BCa biomarker discovery-to capture differentially secreted proteins in BCa patients. We identified five highly sensitive and specific biomarkers, which were validated in urine samples from a large cohort of patients with BCa, benign urological conditions, chronic diseases or other cancer types.

## RESULTS

### Overview of candidate biomarker identification for BCa detection using quantitative MS

To identify potential biomarkers for the diagnosis of Ta/T1 and T2/T3 BCa, we performed MS analysis using pooled urine samples of equal volumes from four subjects. In order to minimize experimental errors, we included biological (different pools) and technical replicates (different MS runs for the same samples). We combined the entire dataset to obtain the mean fold change in proteins present in BCa urine samples as compared with healthy urine samples. Proteins with at least a two-fold and two ratio count difference were considered as over-secreted proteins, and these were then scrutinized through extensive online database literature searching. These proteins were shortlisted to 17 potential hits based on their novelty, previously published cancer association, and subcellular localization.

### Identification of five candidate biomarkers for BCa in voided urine samples

Shortlisted, potential biomarkers were next considered for initial validation by RT-PCR with ten samples for each of the three groups: control (healthy subjects), Ta/T1, and T2/T3 BCa. Based on the results of the mRNA expression analysis (data not shown), we found a higher expression (fold change >2) of five candidate biomarkers-Coronin-1A, Apolipoprotein A4, Semenogelin-2, Gamma Synuclein and DJ-1-in Ta/T1 and T2/T3 BCa samples as compared with samples from healthy subjects (Supplementary Figure 1). The shortlisted potential biomarkers were also validated using western blot analysis of healthy subjects and BCa patient urine samples before determining the five biomarkers.

### Validation of five candidate biomarkers for BCa in a larger cohort

**Figure 1:**
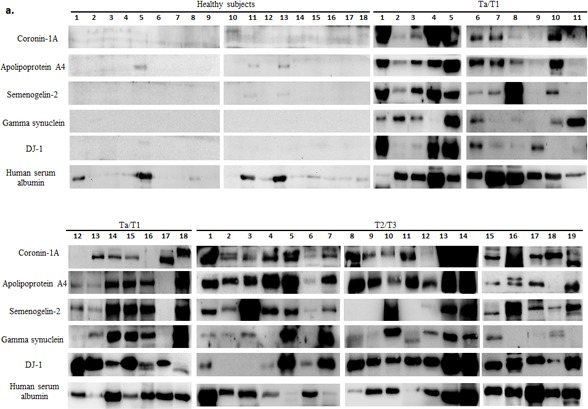

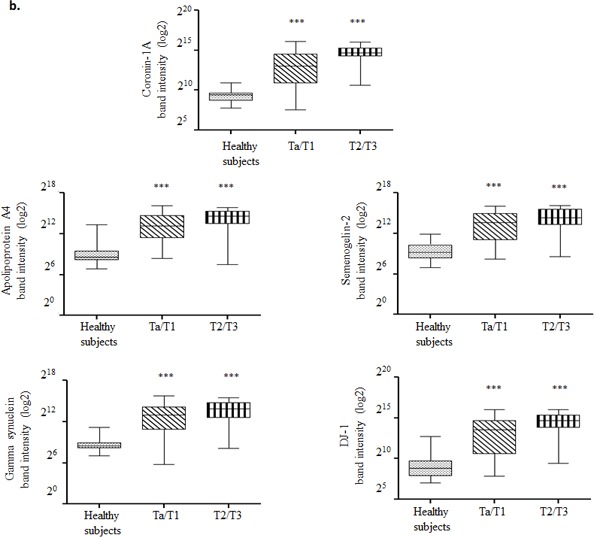
Western blot analysis of urine samples from healthy subjects and bladder carcinoma (BCa) patients **a.** Protein (100 μg) from each voided urine sample of healthy subjects, non-muscle invasive (NMI; Ta/T1) and muscle invasive (MI; T2/T3) BCa patients was subjected to western blotting. Human serum albumin (HSA) protein was also examined in each sample to assess for a correlation between biomarker expression and hematuria. **b.** Box-and-Whisker plot of log2 band intensities of the blots plotted for the five biomarkers from all voided urine samples obtained from healthy subjects (*n* = 66), Ta/T1 (*n* = 110) and T2/T3 (*n* = 63) BCa patients. The box represents the lower quartile, median, and higher quartile values; the whiskers show the minimum and maximum values. Mann-Whitney test was used to compute significance. ***p < 0.001 (as compared with healthy samples).

Urine samples from 66 healthy subjects, 110 Ta/T1 BCa patients and 63 T2/T3 BCa patients were analyzed using western blotting. All BCa urine samples exhibited an elevated level in at least three out of the five biomarkers (Figure 1). ROC curve, established using band intensity values, showed an AUC of 0.98 and 1.0 for healthy versus Ta/T1 and healthy versus T2/T3, respectively, using the five biomarkers in combination (Figure 3; Table 1A and 1B). The data from western blot analyses (Figure 1a and 1b) showed perfect concordance with the RT-PCR data (Supplementary Figure 1), indicating that the five biomarkers were significantly enriched in urine samples of BCa patients as compared with that of healthy subjects. Representative MS spectra (Supplementary Figure 2) of the five potential markers were also in good agreement with RT-PCR and western blotting data.

**Table 1A:** Accuracy of the combination model in diagnosing Ta/T1 bladder cancer

Biomarkers		AUC	Sensitivity %	Specificity %	PPV %	NPV %	Overall accuracy %
ELISA	All five biomarkers in combination	0.92	79.2	100	100	66.7	85.3
Western blot	All five biomarkers in combination	0.98	93.9	96.7	98.4	87.9	94.8
	Three biomarkers (Coronin-1A, Apolipoprotein A4, DJ-1) in combination	0.97	92.4	93.3	96.8	84.8	92.7
	Four biomarkers (Coronin-1A, Apolipoprotein A4, Semanogelin-2, DJ-1) in combination	0.98	90.9	96.7	98.4	82.9	92.7

Abbreviations: AUC, area under the curve; PPV, positive predictive value; NPV, negative predictive value.

**Table 1B:** Accuracy of the combination model in diagnosing T2/T3 bladder cancer

Biomarkers		AUC	Sensitivity %	Specificity %	PPV %	NPV %	Overall accuracy %
ELISA	All five biomarkers in combination	0.94	86.4	100	100	76.9	90.6
Western blot	All five biomarkers in combination	1.0	100	100	100	100	100
	Three biomarkers (Coronin-1A, Apolipoprotein A4, DJ-1) in combination	1.0	100	100	100	100	100
	Four biomarkers (Coronin-1A, Apolipoprotein A4, Semanogelin-2, DJ-1) in combination	1.0	100	100	100	100	100

Abbreviations: AUC, area under the curve; PPV, positive predictive value; NPV, negative predictive value.

### Hematuria had no effect on urinary biomarker levels

Hematuria occurs in numerous patients with urinary tract disease. We wanted to ascertain whether hematuria could affect a BCa diagnosis using our selected biomarkers, as these proteins can also be found in blood; albeit, at low levels. Supplementary Table 5, based on a database search, provides the prevalence and, in some cases, the large concentration range of four of our five biomarkers in plasma. We tested for the expression of these five biomarkers in plasma and in white blood cells from four blood samples using western blot analysis. We found the expression of all five biomarkers in plasma but only Coronin-1A in white blood cell extracts.

**Figure 2:**
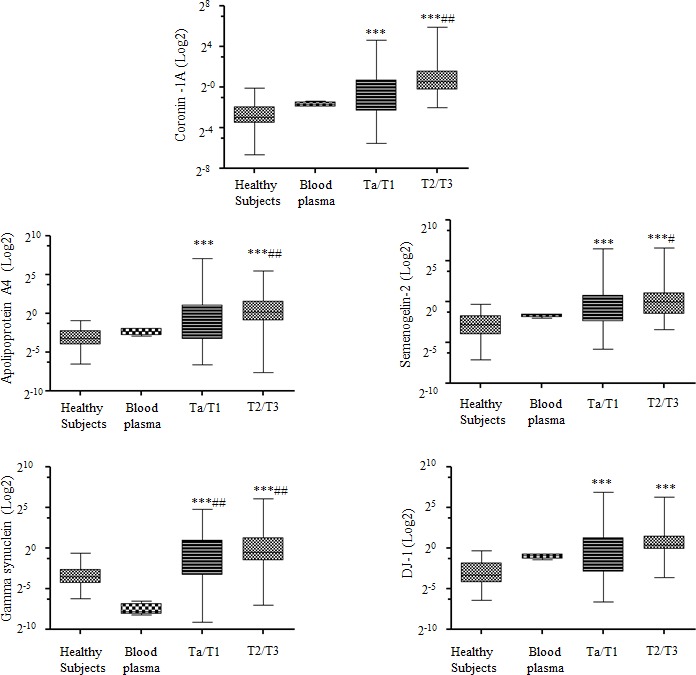
Correlation of biomarkers and hematuria in urine samples Box-and-Whisker plot of the five biomarkers (Coronin-1A, Apolipoprotein A4, Semenogelin-2, Gamma synuclein and DJ-1) with respect to albumin in voided urine samples from healthy subjects (***n*** = 30), blood plasma (***n*** = 4), Ta/T1 bladder carcinoma (BCa) (***n*** = 66), and T2/T3 BCa (***n*** = 28). The box represents the lower quartile, median, and higher quartile values. The whiskers show the minimum and maximum values. Mann-Whitney test was used to compute significance. *p < 0.05, **p < 0.01, ***p < 0.001 as compared with healthy samples; ^#^*p <* 0.05, ^##^*p* < 0.01, ^###^*p* < 0.001 as compared with blood plasma samples.

To compute the correlation of these biomarkers within plasma, we quantified the western blot intensity of the biomarkers in blood and urine samples and normalized them to human serum albumin (HSA) values in each respective sample. The Box-and-Whisker plot shows that all five biomarkers were significantly expressed in Ta/T1 and T2/T3 urine samples relative to that in samples from healthy subjects or in the blood plasma from four individual samples (Figure 2). Moreover, only Coronin-1A showed marginal correlation with HSA levels using a Pearson correlation test (Table 2). From these results, we conclude that our biomarkers were not expressed in urine as a result of hematuria.

**Table 2:** Pearson correlation analysis of blood plasma and white blood cells with biomarkers

Biomarkers	Healthy Subjects	Ta/T1 BCa Samples (non-muscle invasive)	T2/T3 BCa Samples (muscle invasive)
	Pearson *r*	Pearson *r*	Pearson *r*
Coronin-1A	+0.1874	+0.3198	+0.4320
Apolipoprotein A4	+0.4570	−0.1138	+0.1753
Semenogelin-2	+0.3112	+0.1645	+0.2603
Gamma synuclein	+0.3986	−0.035	−0.1208
DJ-1	+0.3198	+0.0092	+0.1300

Pearson correlation coefficient (*r*) for the biomarkers in urine and blood plasma samples from healthy subjects, Ta/T1 BCa (non-muscle invasive), and T2/T3 BCa (muscle invasive) patients. Abbreviations: BCa, bladder carcinoma.

We next sought to correlate Coronin-1A expression with white blood cell count, making the assumption that HSA originated only from blood rather than from defective kidney filtration. We found at least a 289-fold increase in the actual band intensity of Coronin-1A in urine as compared to what could have theoretically originated from white blood cells (see Appendix A). This analysis thus allowed us to exclude any significant contribution of white blood cells to Coronin-1A content.

**Appendix A:** Calculations to demonstrate that the major contributor of Coronin-1A in BCa patient urine is not from lysed WBCs but from the bladder tumor

**Albumin determination**	
Concentration of albumin in blood*	40 μg/μl
Concentration range of albumin in BCa patient urine samples^&^	0.012-2.56 μg/μl
Mean albumin concentration in BCa patient urine samples	1.18 μg/μl
Approximate volume of blood in urine^#^	(1/40)×1.18 = 0.0295 μl
**White Blood Cells (WBC) determination**	
No. of WBC/μl of blood*	~5000 WBCs
Hence, ~0.0295 μl of blood in urine contains	(5000×0.0295) =147.5 WBCs
**Coronin-1A calculation**	
Weight of a single cell*	3.5×10^−3^μg
Amount of protein/cell^§,*^	0.0007 μg
40 μg of lysed WBC from buffy coat would be derived from	(1/0.0007)×40 ≈ 57,000 WBCs^¥^
Coronin-1A blot intensity of 57,000 WBC lysate^£^	34,403
Hence, 147.5 WBCs if lysed in urine would produce intensity of	(34,403/57000)×147.5 = 89.025
Actual mean Coronin-1A intensity from BCa urine sample blot	25,788.58^¶^
Fold-change in intensity for Coronin-1A	(25,788.58/89.025) = 289.68

* Reported in the literature

& Deduced from our experimental analysis; further calculations were done using the higher limit of the range.

# Assumption: Albumin source is solely blood

§ Protein weight is 20% of cells weight

¥ Rounded down for calculations. Actual calculation is 57,142.86 WBCs

£ Result obtained from western blotting experiments conducted in our lab

¶ Averaged value.

### Minimal biomarker prevalence in urine samples from non-bladder cancer patients

To further evaluate the specificity of our biomarkers in BCa, we performed western blotting analysis of 91 urine samples from non-bladder cancer patients and 121 urine samples from patients suffering from diverse chronic ailments (Supplementary Table 1). The vast majority of samples were completely negative for these five biomarkers; albeit, in approximately 5% of the samples, we noticed variable expression of only one or, rarely, two biomarkers (Supplementary Figures 3 and 4).

### Validation of the high sensitivity and specificity of our diagnostic model

**Figure 3:**
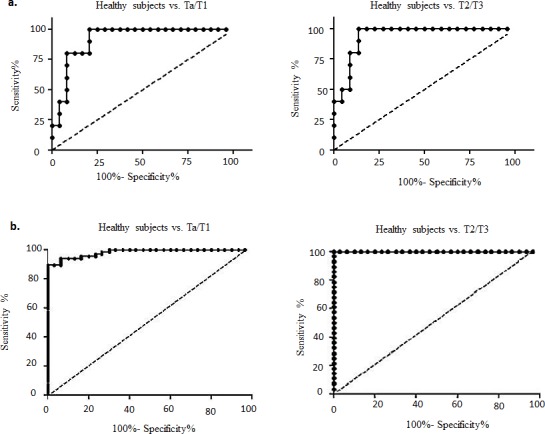
Receiver operating characteristic (ROC) curves designed to evaluate the accuracy of the multiplex biomarker model for the diagnosis of bladder carcinoma (BCa) This model assesses the efficacy of the biomarkers to distinguish both non-muscle invasive (NMI; Ta/T1) and muscle invasive (MI; T2/T3) BCa from healthy subjects based on data obtained from **a.** ELISA analysis and **b.** western blot analysis of voided urine samples.

Finally, we developed a diagnostic model using Lasso regression to evaluate the clinical utility of different combinations of all five urine biomarkers. The diagnostic performances of each of the five biomarkers were computed separately and in various combinations using ELISA and western blotting data (Table 1A and 1B). Sensitivity and specificity, along with the cut-off values, for each biomarker are listed in Supplementary Tables 6A and 6B. Using ELISA expression data, we found that a diagnostic model that combined all five biomarkers was the most accurate, achieving an AUC of 0.92 and an overall accuracy of 85.3% (sensitivity, 79.2%; specificity, 100%) in differentiating Ta/T1 BCa patients from healthy subjects. Similarly, for diagnosing T2/T3 BCa, the diagnostic model achieved an AUC of 0.94 and an overall accuracy of 90.6% (sensitivity, 86.4%; specificity, 100%); as shown in Figure 3 and Tables 1A and 1B. Likewise, using western blotting data, we obtained an AUC of 0.98 and an overall accuracy of 94.8% (sensitivity, 93.9%; specificity, 96.7%) for a Ta/T1 BCa diagnosis, and, surprisingly, an AUC of 1.0 and an overall accuracy of 100% for a T2/T3 BCa diagnosis (Figure 3 and Tables 1A and 1B). Thus, our diagnostic model combining all five biomarkers is highly sensitive and specific, outperforming conventional cytology or any existing biomarkers reported to date for a Ta/T1 BCa diagnosis [13]. Our biomarker panel proved to be superior to the cytology data we obtained from clinics for the healthy subject and BCa patients (Supplementary Table 1).

## DISCUSSION

**Figure 4:**
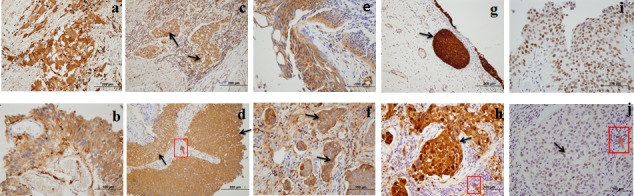
High expression of five biomarkers in bladder carcinoma (BCa) tissue **a.** Clusters of invasive neoplastic cells staining positively for Coronin-1A. **b.** Coronin-1A positively stained carcinoma *in situ* (CIS). **c.** DJ-1 positively stained clusters of invasive carcinoma (black arrow). **d.** DJ-1 positively stained bladder carcinoma (black arrow) and negatively stained inflammatory cells (red arrow). **e.** APOA4 positively stained CIS. **f.** APOA4 positively stained clusters of invasive neoplasm (black arrow). **g.** Gamma-synuclein positively stained CIS (black arrow). **h.** Gamma-synuclein positively stained invasive neoplasm (black arrow) and negatively stained inflammatory cells (red arrow). **i.** Semenogelin-2 positively stained CIS. **j.** Semenogelin-2 positively stained invasive neoplastic cells (black arrow) and negatively stained inflammatory cells (red arrow).

Advanced, MS-based, quantitative proteomics allowed us to identify a panel of novel biomarkers with high precision for BCa diagnosis. The five potential candidates were shortlisted from MS data through extensive published literature filtering followed by RT-PCR, western blotting analyses and ELISA validation. The results showed elevated urinary concentrations of these five biomarkers in patients with BCa, with equally enriched expression found in immunohistochemically stained BCa tissues (Figure 4). Each of these five proteins has an important biological function, as shown by the relevant literature in Supplementary Table 7. Coronin-1A is a crucial component of the actin cytoskeleton and promotes cellular processes including endocytosis and cell motility. It is expressed in brain, thymus, spleen, bone marrow and lymph nodes, and selective cytoplasmic expression has been reported in leukocytes and microglia.

Apolipoprotein A4 (APOA4), a critical mediator of lipid metabolism, is also proposed to be a mediator of reverse cholesterol transport (removes cholesterol from peripheral cells and directs it to the liver for metabolism). In addition, it is regarded as an antioxidant, a mediator of gut inflammation, and has been linked to β-amyloid clearance in the brain. Serum APOA4 is a reported marker of pancreatic [12] and ovarian [14] cancers, and it is differentially expressed in the sera of gastric cancer patients [15]. APOA4 is elevated in patients with chronic kidney disease [16], and APOA1 in diabetic rat bladders [17].

The third biomarker, Gamma synuclein belongs to a family of small, soluble proteins consisting of α-, β- and γ-synuclein. Synucleins have been implicated in neurodegenerative diseases, but the function of γ-synuclein is not completely understood. γ-Synuclein overexpression is known to cause an increase in proliferation, invasiveness and metastasis [18], and γ-synuclein, along with calreticulin and catechol-o-methyltransferase, is reported to be a tumor marker in BCa [19].

Semenogelin-2, the fourth marker, is a major coagulating protein in human semen. Although semenogelin expression is normally restricted to males, two small cell lung carcinoma cell lines from female patients have been reported positive for semenogelin II expression [20]. The role of semenogelins in human malignancy is poorly understood; however, various studies have reported the expression of semenogelins in lung carcinomas, melanoma and leukemias. Semenogelin II has been shown to have prognostic significance in the prediction of cancer progression after radical prostatectomy [21].

Finally, DJ-1 is a conserved protein, coded by the gene Parkinson disease 7 (PARK7). DJ-1 was originally identified as an oncogene that can transform mouse NIH3T3 cells in cooperation with H-RAS [22]. DJ-1 protein affects cell survival by modulating cellular signaling cascades, such as the PTEN–phosphatidylinositol 3-kinase–Akt pathway [23], and can alter the activity of p53 [24]. The elevation of serum DJ-1 levels in patients with pancreatic cancer has led to the identification of DJ-1 as a potential diagnostic marker [25]. DJ-1 expression is correlated with the pathological stages of invasive urothelial carcinoma of the urinary bladder [26].

The diagnostic performance of these five biomarkers indicated a necessity to combine all five for an accurate diagnosis. FDA-approved Complement factor H, which has been reported for the diagnosis of patients with benign renal diseases and urinary tract infections as well as other cancer types, provides only 60% specificity [27]. Our five urine biomarkers were able to differentiate patients with BCa from those with benign conditions (such as inflammation of the bladder, benign prostatic hyperplasia or nephrolithiasis possibly associated with hematuria) with 100% specificity, and were rarely detected in the urine of patients affected by chronic diseases or other cancer types.

The FDA-approved biomarker NMP22 is reported to be affected by hematuria in urine [28]. Hence, we took this issue into consideration during our validation tests. Normal HSA levels in urine vary considerably and can be detected in samples from otherwise healthy subjects. Notably, urine from patients with chronic diseases, such as diabetes, has a much higher level of HSA than that from many of our BCa patients. Therefore, HSA does not always reflect blood contamination. In addition, plasma or white blood cell contamination cannot account for the levels of these biomarkers detected following low-speed centrifugation of urine.

A follow-up study during the course of treatment or with post-treatment patients will be needed to test whether the combination of these five biomarkers can predict patient outcome or detect BCa recurrence. This is particularly critical in patients with superficial BCa who are treated with trans-urethral resection or who undergo Bacillus Calmette-Guerin (BCG) treatment.

## CONCLUSIONS

We established a robust, urinary protein biomarker panel for BCa diagnosis. This panel consists of five biomarkers with significantly higher sensitivity and specificity than that of the currently available, non-invasive diagnostic assays for the detection of BCa. This panel has the potential to be used to not only monitor and follow-up patients with BCa, but also as a tool for screening asymptomatic subjects who are at a high risk of developing BCa. This study is likely to be immensely beneficial to patients as a companion diagnostic in the standard of care or as a potential novel therapeutic.

Non-standard Abbreviations: BCa, bladder cancer; ROC, receiver operating characteristic; MS, mass spectrometry; HSA, human serum albumin; CIS, carcinoma *in situ*.

## MATERIALS AND METHODS

### Urine samples

Voided urine samples and associated clinical information (Supplementary Table 1) were collected following Institutional Review Board approval and informed consent. Samples were obtained from Department of Urology, National University Hospital (IRB #02 235); National University Health System Tissue Repository (IRB B-14-015E); Department of Urology, Singapore General Hospital (IRB 2012-525); Department of Urology, CHU Henri Mondor Creteil, France (IRB 07284); Department of Urology, Hospital Lyon CHU Edouard Herriot Hospital (IRB 2008-073); Department of Urology, Medical School Hamburg, Germany (IRB PV3652) and Department of Urology, Pusan National University Hospital, Pusan, South Korea (IRB PNUH-E-2014118).

For the initial screening, 20 ml of voided urine was collected from healthy subjects and BCa patients in preservation tubes (Norgen Biotek Corporation, Canada) and stored at −80°C. The voided urine was clean-catch, midstream urine, and the samples did not contain squamous cells or microbes. The samples were from three separate groups: the control group, corresponding to healthy subjects with no previous history of urothelial cell carcinoma, gross haematuria, active urinary tract infection or urolithiasis; a second group, formed by Ta/T1 BCa patients, characterized as having non-muscle invasive (NMI) BCa; and a third group, comprising T2/T3 BCa patients, characterized as having muscle invasive (MI) BCa. Each urine aliquot was assigned a unique identifier before laboratory processing. Urine samples were excluded if they had significant blood contamination, as determined by visual inspection and based on the Dip-stick urine test (Combur9, Roche Diagnostics, Basel, Switzerland) [results tabulated in Supplementary Table 2]. Samples were stored at −80°C until further processing. For validation screens, an additional 5 ml of voided urine was collected from patients affected by chronic diseases, BCa, and other cancer types. Control urine samples were obtained from healthy, community-dwelling participants of the Singapore Consortium of Cohort Studies.

### Mass spectrometry analysis

Concentrated urine samples were subjected to on-column stable isotope dimethyl labeling followed by nLC-MS analysis. Urine proteins were concentrated using a 3-kDa centrifugal filter, according to the manufacturer's instructions (Millipore, Carrigtwohill, Ireland). Four samples from each cohort, i.e., healthy, Ta/T1 and T2/T3 BCa, were pooled and centrifuged at 8000 ×*g* for 30–60 min at 4°C. Acetone-precipitated samples were reconstituted in 120 μl of 8 M urea in 100 mM ammonium bicarbonate. Samples were reduced by the addition of 1 M dithiothreitol to a final concentration of 5 mM in each sample, and then incubated at room temperature for 30 min. Alkylation was performed by the addition of 0.5 M iodoacetamide to a final concentration of 10 mM, and the samples were incubated in the dark for 30 min. All samples were then diluted from the initial 8 M urea to 6 M urea using 100 mM ammonium bicarbonate and then incubated with Lys-C (enzyme-to-protein ratio of 1:100) at 37°C overnight. Digestion mixtures were adjusted to a final concentration of 1 M urea by the addition of 50 mM ammonium bicarbonate followed by incubation with trypsin (enzyme-to-protein ratio of 1:50) at 37°C for 4 h.

The tryptic peptide mixtures were subjected to on-column stable isotope dimethyl labelling, as described elsewhere [11], using a triple-labelling approach. Differentially labelled urine samples were mixed and fractionated using isoelectric focusing electrophoresis on an Agilent 3100 OFFGEL Fractionator (12 fractions). The samples were cleaned up using self-packed C18 stage tips and subjected to mass spectrometry analysis, also as previously described [11].

MS data were analyzed by MaxQuant version 1.3.0.5 using the 2013-07_uniprot human FASTA database (88354 entries) [12]. Maximum false discovery rates were set to 0.01 for both protein and peptide. Proteins were considered identified when supported by at least one unique peptide with a minimum length of 7 amino acids.

### Quantitative real-time polymerase chain reaction

Total RNA was isolated from sloughed cells within the 5 ml urine samples using the ZR Urine RNA Isolation Kit (Zymo Research, Irvine, CA). Total RNA (500 ng) was reverse transcribed using SuperScript^®^ III First-Strand Synthesis System (Invitrogen, Carlsbad, CA). RT-PCR pre-amplification reactions were carried out using EXPRESS SYBR^®^ GreenER™ qPCR Supermix with Premixed ROX (Invitrogen). The primer sequences are listed in Supplementary Table 3.

### Western blot analysis

Western blotting was carried out on urine samples after they were centrifuged to eliminate cells. Protein concentrations were estimated using a BCA Protein Assay Kit (Pierce, Rockford, IL). Proteins (100 μg) from individual samples were resolved on sodium dodecyl sulphate (SDS)–polyacrylamide gel electrophoresis (PAGE) and transferred to polyvinylidene difluoride (PVDF) membranes (Bio-Rad Laboratories, Hercules, CA). Membranes were blocked with 5% bovine serum albumin (BSA) in Tris-buffered saline containing 0.1% Tween-20 (TBST) for 1 h at room temperature followed by probing with primary antibodies listed in Supplementary Table 4.

### Protein quantification by ELISA

Urinary protein concentration for each biomarker was determined using respective ELISA kits (USCN Lifescience Inc., Wuhan, Hubei) according to the manufacturer's instructions (in triplicates). The kits are listed in Supplementary Table 4. The biomarker quantity in the urine was normalized by loading an equal concentration of total protein into each well of the ELISA plate.

### Histopathology

BCa biopsy samples were fixed in 10% neutral-buffered formalin for 48 h, transferred to 70% ethanol, and then embedded in paraffin. Tissue sections (4 μm) were stained with hematoxylin and eosin for histopathological examination. Heat-induced epitope retrieval was performed using Bond^TM^ Epitope Retrieval Solution 2 (pH 9.0) for 40 min at 100°C. Immunohistochemical staining was performed using the Leica Bond^TM^ Autostainer, which uses their proprietary Bond^TM^ Detection Refine Kit (Leica, Solms, Germany). All immunofluorescence labelling was performed using antibody dilutions listed in Supplementary Table 4.

### Statistical analysis

Significance evaluation for mean differences and correlations were computed by an unpaired *t*-test and Pearson correlation analysis, respectively, using Matlab^®^ R2012a (MathWorks, Natick, MA). Band intensity from western blots was quantified using ImageJ (National Institutes of Health, Bethesda, MD). A diagnostic model combining the five biomarkers was developed using the Lasso regression model from Matlab^®^ R2012a statistical toolbox version 8.0 (MathWorks, Natick, MA). Calibration curves were prepared using purified proteins provided with the ELISA kits. Receiver Operating Characteristic (ROC) curves and AUC were computed using Graphpad Prism^®^ version 5.0 (GraphPad Software, La Jolla, CA). Thresholds for each biomarker were selected based on Youden's Index. Sensitivity, specificity, positive predictive value (PPV), negative predictive value (NPV), and overall accuracy were computed using the selected threshold for diagnosis. A *P*-value < 0.05 (two-sided) was regarded as significant.

## SUPPLEMENTARY MATERIAL AND TABLES


