# Supplementary material for: Highly sensitive and specific novel biomarkers for the diagnosis of transitional bladder carcinoma
Source: Oncotarget. 2015 Apr 15;6(15):13539–49. doi: 10.18632/oncotarget.3841 (PMC4537032; doi:10.18632/oncotarget.3841)
Supplement: Supplementary file 1 [file oncotarget-06-13539-s001.pdf]

Highly sensitive and specific novel biomarkers for the diagnosis of transitional bladder carcinoma

Supplementary Material

Supplementary Table 1: Healthy Subjects and Patient Urine Sample Details (*n*=451)

Sample type	No. of samples	Clinical stages	Grade	Disease types/ Origin of cancers	Age range (years)	Gender	Cytology results	Volume of urine collected
Healthy Subjects	66	n/a	n/a	n/a	32-75	22F;44M	Positive:0 out of 12 Negative: 12 out of 12	20 ml for initial screening and 5 ml for validation phase
Bladder Cancer patient	110	Ta/T1	Low: 70 High: 40	Bladder cancer	49-86	19F;91M	Positive:5 out of 37 Negative: 32 out of 37	
	63	T2/T3	Low: 19 High: 44		61-87	11F;52M	Positive: 20 out of 44 Negative: 24 out of 44	
Patients with Chronic Ailments	27	n/a	n/a	Diabetes	25-67	-	n/a	5 ml
	27			Hypercholestero-lemia	35-62	-		
	27			Hypertension	20-67	-		
	6			Benign prostatic hyperplasia	35-66	6M		
	4			Bladder inflammation	60-81	3F; 1M		
	4			Nephrolithiasis	56-73	4M		
	1			Pancreatitis	-	-		
	1			Hepatitis	-	-		
	8			Arthritis	-	-		
	9			Asthma	-	-		
	3			Thyroidism	-	-		
	1			Parkinson's disease	-	-		
	1			Gastritis	-	-		
	1			Anemia	-	-		
	1			Uterine fibroids	-	-		
Patients with Various types of Cancer	18	n/a	n/a	Lung	38-70	5F; 13M	n/a	5 ml
	19			Prostate	53-80	19M		
	25			Breast	35-80	25F		
	6			Gastric	24-79	2F; 4M		
	5			Colon	65-74	1F; 4M		
	5			Endometrium	56-77	5F		
	2			Uterus	53-62	2F		
	1			Pancreas	73	1M		
	2			Peritoneum	64-69	2F		
	1			Parotid	43	1M		
	1			Tongue	65	1M		
	2			Thyroid gland	51-76	2F		
	4			Renal	59-65	1F; 3M		

Abbreviation: n/a, not applicable; BCa, bladder carcinoma.

Supplementary Table 2: Roche Combur9 Urine Dipstick Test for the Healthy Subjects and Bladder Cancer Patient Urine Samples

Detection Windows of Combur9 Dipstick Test	Percentage (%) of Samples		
	Healthy Subjects	Ta/T1	T2/T3
Leukocyte			
Negative	56.67	41.67	27.27
10-20 leucocyte/µl (1+)	23.33	44.44	47.27
75 leucocyte/µl (2+)	16.67	8.33	14.55
500 leucocyte/µl (3+)	3.33	5.56	10.91
Nitrite			
Negative	93.33	80.39	83.64
Positive	6.67	19.61	16.36
pH			
5	70	81.94	65.45
6	23.33	13.89	23.64
7	6.67	4.17	10.91
8	-	-	-
9	-	-	-
Protein			
Negative	-	-	-
30 mg/dL (1+)	100	68.06	54.55
100 mg/dL (2+)	-	23.61	29.09
500 mg/dL (3+)	-	8.33	16.36
Glucose			
Normal	96.67	84.94	87.27
50mg/dL (1+)	-	8.33	3.64
100mg/dL (2+)	3.33	1.39	-
300mg/dL (3+)	-	4.17	3.64
1000mg/dL (4+)	-	1.17	5.45
Ketone			
Negative	50	84.72	90.91
10mg/dL (1+)	-	15.28	7.27
50mg/dL (2+)	30	-	1.82
150mg/dL (3+)	20	-	-
Urobilinogen			
Normal	100	94.44	100
1mg/dL (1+)	-	5.56	-
4mg/dL (2+)	-	-	-
8mg/dL (3+)	-	-	-
12mg/dL (4+)	-	-	-
Bilirubin			
Negative	93.33	96.72	97.27
(1+)	6.67	1.89	2.73
(2+)	-	1.39	-
(3+)	-	-	-
Blood			
Negative	79	59.72	61.82
5-10 erythrocytes/µL (1+)	11	11.11	3.64
25 erythrocytes/µL (2+)	10	2.78	14.55
50 erythrocytes/µL (3+)	-	12.5	-
250 erythrocytes/µL (4+)	-	13.89	20

Supplementary Table 3: Primer Sequences for Quantitative Reverse-Transcription Polymerase Chain Reaction (qRT-PCR)

**Genes**	**Forward Sequence (5' → 3')**	**Reverse Sequence (5' → 3')**
Coronin-1A	CTTCAGCCGCATGAGTGAG	AGGTAGACGATGTTGGTGTCA
Apolipoprotein A4	CCCAGCAACTCAATGCCCT	CCTTCAGTTTCTCCGAGTCCT
Semenogelin-2	CCAACATGGACCCAAAGACAT	TGTACGTGAAGACGGGTATGA
Gamma synuclein	CAAGAAGGGCTTCTCCATCGCCAAGG	CCTCTTTCTCTTTGGATGCCACACCC
DJ-1	TGCGTTCACTTTCAGCCT	TGTGACTTCCATACTTCCGC

Supplementary Table 4: Antibodies and ELISA Kits used for Western Blot, Immunostaining and ELISA Assays

Biomarkers	Catalogue No. for Antibodies Used	Western Blot (Antibody Dilution)	Immuno-histochemistry (Antibody Dilution)	ELISA Kits Catalogue no.	Lot No.	ELISA Detection Range
Coronin-1A	NB110-58867	1:5000	1:200	SEJ355Hu	L130704103	0.313-20ng/ml
Apolipoprotein A4	ab59036<br>HPA002549	1:1000	1:300	SEB967Hu	L130609219	4.7-300ng/ml
Semenogelin-2	ab108085	1:1000	1:200	SEH184Hu	L130704101	0.313-20ng/ml
Gamma-synuclein	ab55424	1:1000	1:1300	SEA939Hu	L130704102	0.313-20ng/ml
DJ-1	NB100-483	1:1000	1:600	SEL059Hu	L130704123	1.56-100ng/ml

Supplementary Table 5: Biomarker Concentration in Blood Plasma

Protein	Uniprot Name	Accession Number	Concentration by MS
Coronin-1A	COR1A_HUMAN	P31146	41 ng/ml
Apolipoprotein A4	APOA4_HUMAN	P06727	30 - 50 µg/ml
Semenogelin-2	SEMG1_HUMAN	P04279	6 ng/ml
Gamma synuclein	SYUG_HUMAN	O76070	--
DJ-1	PARK7_HUMAN	Q99497	23 ng/ml

All data are taken from http://www.plasmaproteomedatabase.org/

Supplementary Table 6A: Accuracies and Thresholds of Biomarkers in Ta/T1 Diagnosis using Both ELISA and Western Blot Urine Sample Data Analysis.

Biomarkers		AUC	Threshold (ng/ml) or intensity	Sensitivity %	Specificity %	PPV %	NPV %	Overall Accuracy %
ELISA	Coronin-1A	0.81	2.31	66.7	100	100	55.6	76.5
	Apolipoprotein A4	0.93	0.5	79.2	100	100	66.7	85.3
	Semenogelin-2	0.73	29.1	66.7	80	88.9	50	70.6
	Gamma synuclein	0.88	2.4	87.5	90	95.5	75	88.2
	DJ-1	0.94	2.4	83.3	100	100	71.4	88.2
Western blot	Coronin-1A	0.92	2029.5	75.8	100	100	65.2	83.3
	Apolipoprotein A4	0.93	939.2	87.9	90	95.1	77.1	88.5
	Semenogelin-2	0.91	879.7	92.4	76.7	89.7	82.1	87.5
	Gamma synuclein	0.89	1145.2	81.8	96.7	98.2	70.7	86.5
	DJ-1	0.91	2864.8	74.2	96.7	98	63	81.3

Abbreviation: AUC, area under the curve; PPV, positive predictive value; NPV, negative predictive value.

Supplementary Table 6B: Accuracies and Thresholds of Biomarkers in T2/T3 Diagnosis using Both ELISA and Western Blot Urine Samples Data Analysis.

Biomarkers		AUC	Threshold (ng/ml) or intensity	Sensitivity %	Specificity %	PPV %	NPV %	Overall Accuracy %
ELISA	Coronin-1A	0.85	1.9	81.8	90	94.7	69.2	84.4
	Apolipoprotein A4	1.0	0.5	90.9	100	100	83.3	93.8
	Semenogelin-2	0.79	30.0	77.3	80	89.5	61.5	78.1
	Gamma synuclein	0.79	2.35	72.7	90	95.5	75	88.2
	DJ-1	0.99	2.3	95.5	100	100	90.9	96.9
Western blot	Coronin-1A	0.99	1584.5	100	96.7	96.6	100	98.3
	Apolipoprotein A4	0.95	1681.9	92.9	93.3	92.9	93.3	93.1
	Semenogelin-2	0.94	3947.6	89.3	100	100	90.9	94.8
	Gamma synuclein	0.96	818.9	92.9	96.7	96.3	93.5	94.8
	DJ-1	0.99	2123.4	96.4	93.3	93.1	96.6	94.8

Abbreviation: AUC, area under the curve; PPV, positive predictive value; NPV, negative predictive value.

Supplementary Table 7: Important Biological Function of All Five Biomarkers

Biomarkers	Biological Function
**Coronin-1A**	• Highly expressed in the hematopoietic system and regulates F-actin content in thymocytes [1].<br>• Altered expression in lymphomas and in other hematological malignancies [2].<br>• Identified as a novel antibody target for clinically isolated syndrome and multiple sclerosis.
**Apolipoprotein A-4**	• Synthesized primarily in the intestine and secreted in the plasma.<br>• Plays a central role in lipid absorption, transport, and metabolism within the reverse cholesterol transport pathway and may act as a postprandial satiety signaling factor and as an antioxidant [3].<br>• Associated with various cancer types, including gastric [4], pancreatic [5] and ovarian [6] cancers.
**Gamma synuclein**	• A member of the synuclein family of proteins, which also comprises alpha and beta subtypes.<br>• Found in advanced breast, ovarian, gastric, esophageal, liver, colon, pancreatic, and bladder cancers [7].<br>• Promotes cancer invasion and metastasis both *in vitro* and in animal models [8].
**Semenogelin-2**	• Secreted protein involved in liquefaction of the human semen coagulum and the progressive release of motile spermatozoa A [9].<br>• Detected in small cell lung carcinoma cell lines [10].
**DJ-1**	• Protein product of *PARK7*.<br>• Positively regulates androgen receptor-dependent transcription.<br>• Overexpressed and correlated with tumor progression in breast, lung, blood, prostate, cervix, thyroid and pancreas malignancies [11].

[1] N. Foger, L. Rangell, D. M. Danilenko, A. C. Chan. Requirement for coronin 1 in T lymphocyte trafficking and cellular homeostasis. Science. 2006;313:839-842.
[2] C. S. Wilson, G. S. Davidson, S. B. Martin, E. Andries, J. Potter, R. Harvey, et al. Gene expression profiling of adult acute myeloid leukemia identifies novel biologic clusters for risk classification and outcome prediction. Blood. 2006;108:685-696.
[3] X. Deng, J. Morris, J. Dressmen, M. R. Tubb, P. Tso, W. G. Jerome, et al. The structure of dimeric apolipoprotein A-IV and its mechanism of self-association. Structure. 2012;20:767-779.
[4] W. Liu, B. Liu, Q. Cai, J. Li, X. Chen, Z. Zhu. Proteomic identification of serum biomarkers for gastric cancer using multi-dimensional liquid chromatography and 2D differential gel electrophoresis. Clin Chim Acta. 2012;413:1098-1106.
[5] M. Abulaizi, T. Tomonaga, M. Satoh, K. Sogawa, K. Matsushita, Y. Kodera, et al. The application of a three-step proteome analysis for identification of new biomarkers of pancreatic cancer. Int J Proteomics. 2011;2011:628787.
[6] H. Dieplinger, D. P. Ankerst, A. Burges, M. Lenhard, A. Lingenhel, L. Fineder, et al. Afamin and apolipoprotein A-IV: novel protein markers for ovarian cancer. Cancer Epidemiol Biomarkers Prev. 2009;18:1127-1133.
[7] J. Chen, L. Jiao, C. Xu, Y. Yu, Z. Zhang, Z. Chang, et al. Neural protein gamma-synuclein interacting with androgen receptor promotes human prostate cancer progression. BMC Cancer. 2012;12:593.
[8] T. Jia, Y. E. Liu, J. Liu, Y. E. Shi. Stimulation of breast cancer invasion and metastasis by synuclein gamma. Cancer Res. 1999;59:742-747.

[9] K. Yoshida, Z. T. Krasznai, Z. Krasznai, M. Yoshiike, N. Kawano, M. Yoshida, et al. Functional implications of membrane modification with semenogelins for inhibition of sperm motility in humans. Cell Motil Cytoskeleton. 2009;66:99-108.
[10] R. G. Rodrigues, A. Panizo-Santos, J. A. Cashel, H. C. Krutzsch, M. J. Merino, D. D. Roberts. Semenogelins are ectopically expressed in small cell lung carcinoma. Clin Cancer Res. 2001;7:854-860.
[11] D. A. Hinkle, S. J. Mullett, B. E. Gabris, R. L. Hamilton. DJ-1 expression in glioblastomas shows positive correlation with p53 expression and negative correlation with epidermal growth factor receptor amplification. Neuropathology. 2011;31:29-37.
